# Reverse Septal Movement: A Step Forward in the Comprehension of the Underlying Causes

**DOI:** 10.3390/jcm13040928

**Published:** 2024-02-06

**Authors:** Enrico Emilio Diviggiano, Sara Rosi, Federico Landra, Carmine Marallo, Cristina Scoppa, Debora Castellani, Giulia Elena Mandoli, Maria Concetta Pastore, Luna Cavigli, Flavio D’Ascenzi, Matteo Cameli, Marta Focardi

**Affiliations:** 1 Department of Medical Biotechnologies, Division of Cardiology, University of Siena, 53100 Siena, Italy; sara.rosi@student.unisi.it (S.R.); f.landra@student.unisi.it (F.L.); carmine.marallo@gmail.com (C.M.); giulia_elena@hotmail.it (G.E.M.); mariaconce.pastore@unisi.it (M.C.P.); luna.cavigli2@unisi.it (L.C.); flavio.dascenzi@unisi.it (F.D.); matteo.cameli@unisi.it (M.C.); focardim@hotmail.com (M.F.); 2 Department of Medical Biotechnologies, University of Siena, 53100 Siena, Italy; cristinascoppacv@gmail.com (C.S.); debora.castellani@unisi.it (D.C.)

**Keywords:** coronary artery bypass surgery, reverse septal movement, heart failure, speckle tracking, left ventricular remodeling

## Abstract

(1) **Background:** Reverse septal movement (RSM) often occurs after cardiac surgery, consisting of a paradoxical systolic movement of the interventricular septum. In this retrospective study, we aimed to investigate possible determinants of RSM after coronary artery bypass surgery (CABG). (2) **Methods**: Patients who underwent CABG with on- or off-pump techniques at our center from March 2019 to October 2021 were retrospectively included. Exclusion criteria were: exposure to combined procedures (e.g., valve implantation), prior cardiac surgery, intraventricular conduction delays, and previous pacemaker implantation. Laboratory tests and echocardiographic and cardiopulmonary bypass (CPB) duration data were collected. (3) **Results**: We enrolled 138 patients, of whom 32 (23.2%) underwent off-pump CABG. Approximately 89.1% of the population was male; the mean age was 70 ± 11 years. There was no difference in RSM incidence in patients undergoing the off-pump and on-pump techniques (71.9% vs. 62.3%; *p* = 0.319). In patients undergoing on-pump surgery, the incidence of RSM was slightly higher in longer CPB procedures (OR 1.02 (1.00–1.03) *p* = 0.012), and clamping aortic time was also greater (OR 1.02 (1.00–1.03) *p* = 0.042). (4) **Conclusions**: CPB length seems to be correlated with a higher RSM appearance. This better knowledge of RSM reinforces the safety of CABG and its neutral effect on global biventricular function.

## 1. Introduction

Reverse septal movement (RSM) or septal bounce is characterized by a systolic paradoxical movement of the interventricular septum (IVS) with a normal thickening that is displaced towards the right ventricular cavity, followed by a jerky movement towards the left ventricle in early diastole.

This phenomenon is frequently observed after cardiopulmonary bypass (CPB) and after uncomplicated cardiac surgery in general; the reported incidence ranges from 40% to 100% [1,2]. It is also wrongly referred to in clinical practice as post-CPB IVS movement.

It could be easily diagnosed by 2D echocardiography focusing on IVS systolic motion or by using M-mode in parasternal long-axis view with the ultrasound beam perpendicular to the septum and noticing an almost sinuous movement of the septum in monoplanar vision.

RSM usually appears immediately after surgery and, in some cases, tends to reduce or disappear over time, even if it may be persistent indefinitely in some patients. This characteristic movement usually does not affect the overall right and left ventricular (LV) function [3,4,5]. The LV function, in fact, has an obvious clinical relevance in these patients, in which mildly to severe reduction of the ejection fraction (EF) is not rare. However, RSM could gain functional relevance in cases of moderate-to-severely reduced left ventricular ejection fraction (EF).

The pathophysiological mechanisms underlying RSM are not explained yet. A longer CPB time could enhance an inflammatory state [6,7], the release of cytokines, and other factors that could affect IVS myocardiocyte contraction. Many times, RSM was addressed for ischaemia, but studies involving cardiac magnetic resonance ruled out this option in surgical patients [8]. 

There are many other clinical conditions mimicking RSM, such as left bundle branch block, mitral stenosis, pericardial diseases, and ischaemia itself [9]. 

The aim of this retrospective study was to investigate if CPB during cardiac surgery could be correlated with a higher incidence of RSM and, in particular, if CPB duration could be considered a strong determinant of RSM in patients undergoing on-pump CABG. This understanding will give a better understanding of this phenomenon and will improve its understanding in the clinical field in terms of the development and consequences of RSM. There could be a novel approach to RSM for two reasons: there is no need to prevent its onset as far as cardiovascular prognosis is concerned, and, on the other hand, the clinician could fully explain and reassure patients about the safe implication of RSM in cardiac function.

## 2. Materials and Methods

In this single-center retrospective observational study, consecutive patients who underwent CABG for obstructive coronary artery disease alone between March 2019 and October 2021 at our department were enrolled, independently from the use of peri-surgical CPB. Exclusion criteria were: exposure to combined procedures (e.g., valve implantation, ascending aorta surgery), prior cardiac surgery, intraventricular conduction delays, and previous pacemaker implantation. All the anthropometric and anamnestic data were collected. This study did not involve an emergency CABG but only scheduled surgical procedures for previously evaluated patients. All work was in compliance with the 1975 Declaration of Helsinki and was approved by the local ethical commission (protocol n 23440, 12/2022).

### 2.1. Basic Echocardiography

Echocardiographic examination was performed the day before surgery and within 72 h after surgery according to the European Association of Cardiovascular Imaging /American Society of Echocardiography (EACVI/ASE) recommendations for chamber quantification [10], using the high-quality ultrasound machine Vivid iq (GE Medical System, Horthen, Norway). Identification of RSM was performed independently by a single investigator with an advanced level in echocardiography blinded to baseline and procedural data; it was classified in a conventional visual and thus qualitative way. RSM was graded as follows: 0 in the presence of a normal septal motion; 1 if the interventricular septum (IVS) was displaced towards the right ventricular cavity, followed by a jerky movement towards the left ventricle in early diastole.

LA dimensions were calculated from the apical 4-chamber view, and LA volume was indexed for the body surface area (BSA), obtaining the LA volume index (LAVI). LV dimensions were estimated from a long-axis parasternal view in 2D mode. LV ejection fraction (LVEF) was evaluated using the biplane-modified Simpson method from the apical 4- and 2-chamber views. From the 4-chamber view, maximum early diastolic (E) and late diastolic (A) velocities were assessed by trans-mitral pulsed wave doppler; then, peak early diastolic (E’) annular velocities were obtained by tissue doppler imaging, and the E/E’ ratio was calculated and used as an index of the LV filling pressure. Right sections of the heart were assessed through tricuspid annular plane systolic excursion (TAPSE) measured by M-mode, peak systolic (S’) annular velocities obtained by tissue doppler imaging, and fractional ventricular area change (RVFAC). Mitral and tricuspid regurgitation (MR, TR) were quantified by 2D echocardiography according to EACVI/ASE recommendations [10].

### 2.2. Speckle Tracking Echocardiography

Speckle tracking echocardiography (STE) analysis was performed on apical 2-, 3-, and 4-chamber images obtained by 2D gray-scale echocardiography with a stable electrocardiographic recording. Measurements from three consecutive heart cycles were recorded and averaged. The frame rate was 60–80 frames/s. Analysis was performed off-line by a single experienced and independent echocardiographer, who was not directly involved in the image acquisition and blinded to basic echocardiographic parameters, using semiautomated 2D-strain software (EchoPac, GE, Version 204, Milwaukee, WI, USA). The endocardial border was first traced manually, and then the software elaborated the region of interest (ROI) of six segments for each view; afterward, longitudinal strain curves for each segment were generated. LV global longitudinal strain (GLS) was calculated as the mean value of 4-, 2-, and 3-chamber longitudinal strain curves. The global RV longitudinal strain was obtained as the average strain of the RV-free wall and interventricular septum segments from an apical 4-chamber view. Free-wall RV longitudinal strain (fwRVLS) was the result of an ROI comprised of 3 segments that the software elaborated and displayed as 3 curves, corresponding to the strain of each RV free-wall segment plus a dashed curve showing the average value of the previous curves, which was used to calculate fwRVLS.

### 2.3. Blood Testing

We collected a complete routine blood test performed on the same day of the post-surgical echocardiographic evaluation, including blood count (e.g., erythrocytes, hemoglobin, hematocrit, platelets), along with kidney and liver function (e.g., creatinine, bilirubin, oxaloacetic and pyruvic transaminases, total protein). In order to go deeper into a possible systemic or ischemic etiology, we made a broader evaluation involving inflammatory response markers and myocardial injury markers (e.g., C-reactive protein, Hs troponin) [11,12].

### 2.4. Statistical Analysis

Categorical variables were expressed as numbers and percentages. Normally distributed continuous variables were expressed as mean ± Standard Deviation (SD), while those not normally distributed were expressed as the median and interquartile range, where the normal distribution of values was verified with the Kolmogorov-Smirnov test.

The chi-square test was used to compare categorical variables, while the Student’s *t* test, for independent variables, was used to compare continuous variables.

Binary logistic regression was used so as to verify the predictive capacity of some baseline variables with respect to the outcome/dependent variable, namely RSM development. This analysis was performed to provide a potential mechanistic insight into RSM development.

The sample size for the present study was not statistically defined but imposed by the availability of complete echocardiographic examinations to be reviewed in the selected time period.

Statistical analysis was conducted using SPSS Statistics software version 26 (SPSS Inc., Chicago, IL, USA). A *p*-value less than 0.05 was considered statistically significant.

## 3. Results

Among the 271 screened patients, 138 were finally enrolled in this study. We excluded 133 subjects due to exposure to combined procedures (58), previous heart surgery (13), the presence of intraventricular conduction blocks (41), and pacemakers (21). Regarding the surgery technique, 31 (22.5%) patients underwent off-pump CABG, and 107 (77.5%) underwent on-pump CABG. The study population was then divided into two groups according to RSM occurrence. The majority was represented by men (123; 89.1%). The median weight was 78 kg (70–86), the mean BMI was 27.7 kg/m^2^ ± 4.3, the median height was 170 cm (168–175), and the median BSA was 1.9 m^2^ (1.7–2.1). There were no differences in these parameters between the two groups. 

### 3.1. Surgery Features

The CPB duration was 110 ± 34 min, and the median aortic clamping time was 82 min (67–106) in patients who underwent on-pump surgery. Slightly longer CPB was observed in patients who developed RSM (112 min (90–139) vs. 94 min (73–124), *p* 0.010), and the aortic clamping time was slightly longer (87 min (68–110) vs. 70 min (54–105), *p* 0.046).

### 3.2. Laboratory Results

No differences in the collected laboratory parameters were observed (e.g., blood count, electrolytes, C-reactive protein) between patients with and without RSM. The high-sensitivity troponin level was similar in the two groups of patients after surgery, as shown in Table 1. A mild anemia was also present in both groups without a significant difference. Kidney function was substantially overlapping in the off-pump and on-pump groups according to creatinine values. This similarity was also found regarding the total protein level.

### 3.3. Echo and Longitudinal Strain Analysis

The incidence of RSM in patients undergoing the off-pump and on-pump techniques was comparable (71.9% vs. 62.3%, respectively; *p* = 0.319). 

**Figure 1:**
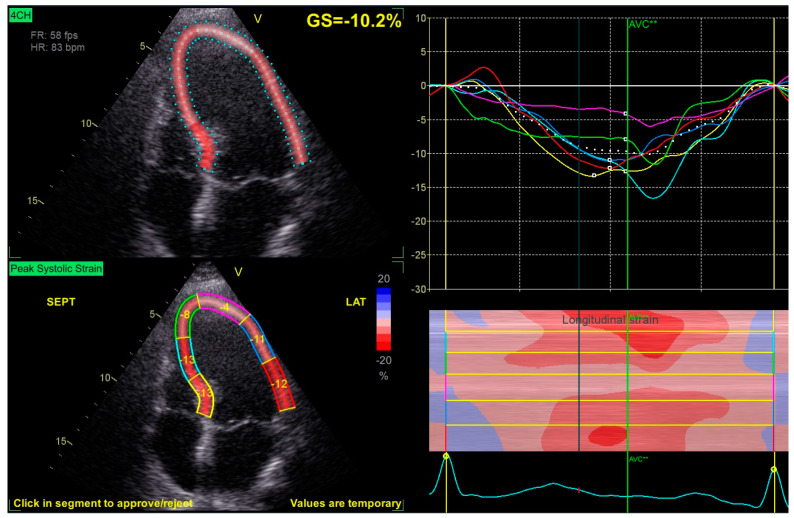
Left Ventricular 4-Chamber Longitudinal Strain Evaluation. AVC = aortic valve closure, FR = frame rate, GS = global strain, HR = heart rate, LAT = lateral, SEPT = septal.

STE-derived LV-GLS in the post-operative echocardiogram was similar between the two groups, as was LVEF before and after surgery, showing a negligible impact of RSM on the global function of the chamber (Figure 1). 

RV-GLS and fwRVLS were reduced along with the other indices of RV longitudinal function. On the other hand, there was a preserved RV global function in the vast majority of cases, as assessed by RVFAC, which considers the effective global contraction of the right ventricle (Table 2).

### 3.4. Prediction of RSM Development

From the univariate regression analysis, the only predictors of RSM were aortic clamping time and CPB duration (see Table 3); for each additional minute of aortic clamping time and CPB, there was a 2% higher probability of developing RSM.

## 4. Discussion

Along with previous studies on this topic, the incidence of RSM was similar in our population of patients undergoing CABG by on-pump and off-pump techniques. However, we found that, in the on-pump group, a higher duration of CPB and a longer aortic clamping duration were related to a higher probability of developing RSM. Actually, longer CPB time could enhance the inflammatory state [6,7], and the release of cytokines, enzymes, or other factors could affect IVS myocardiocyte contraction.

Alongside, there was no significant variation in the systolic function evaluated through the ejection fraction (EF), so the RSM does not affect global LV function in these terms, as we can assume in comparing LV GLS in patients who develop or do not have the RSM. 

After surgery, there was a decrease in regional RV longitudinal shortening (TAPSE), but RV fractional area change (RVFAC) remained normal. It seems likely that RSM is related to the altered regional RV contractile patterns that preserve global RV performance [3,4,5].

In addition, there was no significant difference in the LAVI and E/E′ ratios between the two groups of patients after surgery, thus there is no diastolic function impairment following bypass in either way.

The population of the study was mainly made up of overweight men, by virtue of the fact that obesity and metabolic syndrome, in general, are among the risk factors for cardiovascular pathologies. By comparing the weight and BSA variables in the population with and without RSM, it emerged that the population that developed RSM was predominantly obese or overweight, even if the difference between the two groups was non-significant. 

Pericardial surgery could also be responsible for reverse septal motion [9,13,14]. There could be an unbalance of intraventricular pressures, and in particular, the second establishes a new mechanical environment in which the heart could work only through a new system of forces that could limit heart-filling pressures, or on the other side, there could be less rest and support for the heart, a situation we can assume is closer to the congenital absence of the pericardium. 

CPB has been related to enzyme release with the following biochemical changes: altered right ventricular functioning, mechanical changes (reduced left ventricular basal anteroseptal segment rotation), and right ventricular shape changes becoming more spherical [3,4,15].

A study by Reynolds et al. [2] considered more than three thousand cardiac surgeries, including coronary artery bypass surgery, in which it was concluded that myocardial ischemia can play an important role in the genesis of PSM (Paradoxical Septal Motion), given that increasing clamping times also increased the incidence of this event, as we confirmed with our results. According to this study, patients undergoing off-pump CABG were less likely to develop RSM after surgery, consistent with the concept that ischemia is a contributing factor to paradoxical septal motion. However, many times RSM was addressed to ischaemia in the past [2], in after-CABG patients, and also in the broader field of patients who underwent cardiac surgery, but studies involving heart magnetic resonance ruled out this option in after-surgery patients [8], and in line with this assertion, there was no significant difference between high-sensitivity troponin levels in patients with and without RSM (see Table 3). Furthermore, considering that RSM has also been found in off-pump surgeries, the aortic clamping time is not a necessary and sufficient condition for RSM to occur. The most probable hypothesis is that the pathogenesis is multifactorial, but a precise cause has not yet been identified.

The laboratory data showed a low level of proteins in patients after CABG given by the hemodilution induced during CPB. Yet, as can be seen from the results, this does not influence the outcome from an inflammatory response point of view; actually, it can also be observed in patients undergoing off-pump surgery as well. A mild anemia was also seen postoperatively, which could be due to perioperative blood loss, inflammatory cytokine production, and inadequate fluid administration. Finally, a slight leukocytosis, mostly neutrophil granulocytes, was observed in our patients, probably in response to surgical stress and to interactions of blood with extra-organic material when CPB was deployed. Thus, laboratory values were in line with what is already known about a heart surgical procedure; there was a process of hemodilution, with a low inflammatory activation along with preserved renal function and a mild necrosis index due to the mechanical stress.

### Limitations

First of all, the retrospective design of this study brings well-known limitations, potentially limiting both internal and external validity of this study because of the difficulty in controlling for sources of common biases (i.e., selection bias). However, observation bias was limited by keeping the echocardiographic examiners blinded to baseline and procedural data. The result could be weakened, probably because of the effects of several confounding elements correlated to cardiac surgery, such as the technique, cardioplegic solution composition, cardioplegic duration, the bypassed coronary artery, how many coronary arteries are involved, and the health status of the graft employed. None of the analyzed parameters was able to predict with sufficient significance the occurrence of RSM.

## 5. Conclusions

In the present study, it can be seen that in patients undergoing the on-pump technique, a higher exposure to CPB times was observed in those who developed RSM, along with a higher aortic clamping time. However, considering that RSM was found in off-pump surgeries, the aortic clamping time is not a necessary and sufficient condition for RSM to occur. The most probable hypothesis at the moment is that the pathogenesis is multifactorial, and a precise cause has not yet been identified. Further insights about RSM physiopathology and etiology would require a wider multicentric study with the use of a post-surgical multimodality imaging approach, including, for example, a perfusion exam (e.g., cardiac SPECT) to evaluate possible ischemic damage and cardiac magnetic resonance to describe possible septal early edema or late fibrosis.

## Figures and Tables

**Table 1:** Anatomic features and Laboratory test results.

Characteristic	All (n = 138)	RSM (n = 89)	No RSM (n = 49)	*p*-Value
Age (years)	70.0 ± 11.0	70.0 ± 10.0	70.0 ± 13.0	0.705
Male (%)	123 (89.1)	79 (88.8)	44 (89.8)	
BSA (m^2^)	1.9 (1.7–2.1)	1.9 (1.8–2.1)	1.9 (1.8–1.9)	0.079
Weight	78.0 (70.0–86.0)	80.0 (70.0–90.0)	75.0 (70.0–81.0)	0.072
Hemoglobin	10.7 (9.9–11.6)	10.7 (10.1–11.8)	10.8 (9.7–11.6)	0.889
Leukocytes	10.4 (8.3–4.7)	11.6 (8.4–14.7)	12.0 (8.1–14.7)	0.639
Proteins	4.7 ± 0.7	4.7 ± 0.6	4.8 ± 0.8	0.899
Hs Tn	248.0 (153.0–448.0)	265.0 (175.0–496.0)	214.0 (84.0–307.0)	0.064
Creatinine	0.9 (0.7–1.2)	0.9 (0.7–1.2)	0.9 (0.8–1.2)	0.645
LDH	228.0 (185.0–274.0)	220.0 (183.0–271.0)	220.0 (183.0–271.0)	0.124
Potassium	4.1 ± 0.5	4.1 ± 0.4	4.1 ± 0.6	0.787

RSM = reverse septal movement BSA = body surface Area, Hs Tn = high sensitivity troponin, LDH = lactate dehydrogenase.

**Table 2:** Echocardiogram features in patients with and without RSM after surgery.

Characteristic	All (n = 138)	RSM (n = 89)	No RSM (n = 49)	*p*-Value
LAVI	36 (29–43)	33 (26–42)	39 (32–46)	0.705
IVS	12 (11–13)	12 (11–13)	12 (11–13)	0.852
LVEF	55 (45–55)	55 (45–55)	50 (45–55)	0.977
LV ED velocity cm/s	74 ± 17	73 ± 17	75 ± 14	0.543
LV AD velocity cm/s	74 (60–89)	75 ± 18	71 ± 14	0.985
LV EDm. TDI, cm/s	8 (7–9)	8 (7–10)	8 (7–9)	0.760
E/E’ ratio	9 (7–11)	9 (7–11)	9 (8–12)	0.567
TAPSE	13 (11–15)	13 (11–16)	13 (11–15)	0.079
RV Sm, TDI, cm/s	9 (9–11)	9 (8–11)	9 (8–11)	0.072
RV mEDD, mm	30 (28–32)	30 (27–32)	30 (28–33)	0.889
RV FWS	−13.3 ± 5.1	−16 ± 5.0	−14.2 ± 2.8	0.002
RV GLS	−13 ± 5	−13 ± 5	−14 ± 3	0.639
RVFAC	34 ± 12	34 ± 13	36 ± 8	0.642
LV APLAX LS	−10 ± 3	−10 ± 3	−9 ± 2	0.900
LV 4Ch LS	−10 ± 3	−10 ± 3	−10 ± 2	0.923
LV 2Ch LS	−9 (−12–−7)	−10 (−14–−8)	−8 (−11–−7)	0.791
LV GLS	−11 (−12–−8)	−11 (−12–−8)	−10 (−11–−9)	0.917

LAVI = left atrial volume index, IVS = interventricular septum, TAPSE = tricuspid annular plane systolic excursion, RV = right ventricular, Sm = systolic myocardial, EDm = early diastolic myocardial, TDI = tissue doppler imaging, LV = left ventricular, mEDD = middle-end dastolic diameter, GLS = global longitudinal strain, RV FWS = RVFAC = right ventricular fractional area change, APLAX = apical 3 chamber view, 4Ch = 4 chamber view, 2Ch = 2 chamber view.

**Table 3:** Univariate analysis is used for the prediction of RSM development.

Parameters	RSM Development	*p*-Value
BSA (m^2^)	OR 7.92 (0.76–12.33)	0.083
Weight	OR 1.03 (0.99–1.06)	0.072
Post-surgical LAVI	OR 0.97 (0.94–0.99)	0.056
CPB time	OR 1.02 (1.00–1.03)	0.012
Aortic clamping time	OR 1.02 (1.00–1.03)	0.042

BSA = body surface Area, LAVI = left atrial volume index, CPB = cardiopulmonary bypass.

## Data Availability

Data will be available upon request.

## References

[1] Stanley A., Athanasuleas C., Nanda N. (2022). Paradoxical Septal Motion after Uncomplicated Cardiac Surgery: A Consequence of Altered Regional Right Ventricular Contractile Patterns. Curr. Cardiol. Rev..

[2] Reynolds H.R., Tunick P.A., Grossi E.A., Dilmanian H., Colvin S.B., Kronzon I. (2007). Paradoxical septal motion after cardiac surgery: A review of 3292 cases. Clin. Cardiol..

[3] AHedman A., Alam M., Zuber E., Nordlander R., Samad B.A. (2004). Decreased Right Ventricular Function after Coronary Artery Bypass Grafting and Its Relation to Exercise Capacity: A Tricuspid Annular Motion-based Study. J. Am. Soc. Echocardiogr..

[4] Roshanali F., Yousefnia M.A., Mandegar M.H., Rayatzadeh H., Alinejad S. (2008). Decreased right ventricular function after coronary artery bypass grafting. Tex. Heart Inst. J..

[5] Kang M.-K., Chang H.-J., Cho I.J., Shin S., Shim C.-Y., Hong G.-R., Yu K.-J., Chang B.-C., Chung N. (2014). Echocardiographic investigation of the mechanism underlying abnormal interventricular septal motion after open heart surgery. J. Cardiovasc. Ultrasound.

[6] Raja S.G., Dreyfus G.D. (2005). Modulation of Systemic Inflammatory Response after Cardiac Surgery. Asian Cardiovasc. Thorac. Ann..

[7] Bruins P., te Velthuis H., Yazdanbakhsh A.P., Jansen P.G., van Hardevelt F.W., de Beaumont E.M., Wildevuur C.R., Eijsman L., Trouwborst A., Hack C.E. (1997). Activation of the complement system during and after cardiopulmonary bypass surgery: Postsurgery activation involves c-reactive protein and is associated with postoperative arrhythmia. Circulation.

[8] Rösner A., Avenarius D., Malm S., Iqbal A., Schirmer H., Bijnens B., Myrmel T. (2015). Changes in Right Ventricular Shape and Deformation Following Coronary Artery Bypass Surgery-Insights from Echocardiography with Strain Rate and Magnetic Resonance Imaging. Echocardiography.

[9] Clancy D.J., Mclean A., Slama M., Orde S.R. (2018). Paradoxical septal motion: A diagnostic approach and clinical relevance. Australas. J. Ultrasound Med..

[10] Lang R.M., Badano L.P., Mor-Avi V., Afilalo J., Armstrong A., Ernande L., Flachskampf F.A., Foster E., Goldstein S.A., Kuznetsova T. (2015). Recommendations for cardiac chamber quantification by echocardiography in adults: An update from the American society of echocardiography and the European association of cardiovascular imaging. Eur. Heart J. Cardiovasc. Imaging.

[11] Takaki S., Shehabi Y., Pickering J.W., Endre Z., Miyashita T., Goto T. (2015). Perioperative change in creatinine following cardiac surgery with cardiopulmonary bypass is useful in predicting acute kidney injury: A single-centre retrospective cohort study. Interact. Cardiovasc. Thorac. Surg..

[12] Cardiopulmonary Bypass. www.cambridge.org.

[13] De Nardo D., Caretta Q., Mercanti C., Alessandri N., Scibilia G., Chiavarelli R., Antolini M., Pitucco G., Caputo V., Marino B. (1989). Effects of uncomplicated coronary artery bypass graft surgery on global and regional left ventricular function at rest. Study by equilibrium radionuclide angiocardiography. Cardiology.

[14] Waggoner A.D., Shah A.A., Schuessler J.S., Crawford E.S., Nelson J.G., Miller R.R., Quinones M.A. (1982). Effect of cardiac surgery on ventricular septal motion: Assessment by intraoperative echocardiography and cross-sectional two-dimensional echocardiography. Am. Heart J..

[15] Codreanu I., Pegg T.J., Selvanayagam J.B., Robson M.D., Rider O.J., Dasanu C.A., Jung B.A., Taggart D.P., Clarke K., Holloway C.J. (2011). Details of left ventricular remodeling and the mechanism of paradoxical ventricular septal motion after coronary artery bypass graft surgery. J. Invasive Cardiol..

